# RNA 5-Methylcytosine Modification in Myocardial Fibrosis

**DOI:** 10.31083/RCM44079

**Published:** 2025-10-28

**Authors:** Bohan Li, Yan Hao, Wendan Tian, Wei Liu

**Affiliations:** ^1^Department of Clinical Medicine, Harbin Medical University, 150001 Harbin, Heilongjiang, China; ^2^Department of Cardiology, Weifang People’s Hospital, 261000 Weifang, Shandong, China; ^3^Department of Cardiology, Heilongjiang Provincial Hospital, 150001 Harbin, Heilongjiang, China; ^4^Department of Geriatric Cardiovascular Disease, Guangdong Provincial People’s Hospital, 510080 Guangzhou, Guangdong, China

**Keywords:** m5C methylation, immunoinflammation, myocardial fibrosis, epigenetic modification, RNA modification

## Abstract

Myocardial fibrosis represents a common pathological hallmark of various cardiovascular diseases progressing to heart failure, with the immunoinflammatory response playing a pivotal role in the pathogenesis of myocardial fibrosis. Accumulating evidence suggests that the immune microenvironment modulates myocardial fibrosis by regulating RNA epigenetic modifications, with 5-methylcytosine (m5C) methylation emerging as a key player in this process. This review systematically summarizes the characteristics of m5C methylation modification, the regulatory enzymes involved, and their biological functions in immunoinflammatory responses and myocardial fibrosis. Furthermore, this review examines the molecular mechanisms underlying m5C methylation-mediated regulation of myocardial fibrosis, encompassing the activation of immune cells, the transdifferentiation of cardiac fibroblasts, and the regulation of collagen metabolism. Moreover, the potential clinical implications of targeting m5C methylation for treating myocardial fibrosis are discussed, with an emphasis on future therapeutic prospects.

## 1. Introduction

Heart failure, as the terminal stage of various cardiovascular diseases, is characterized by high morbidity and mortality that are comparable to those of malignant tumors, thereby significantly impairing patients’ quality of life [1, 2, 3]. Its high treatment costs and repeated hospitalizations impose a heavy burden on patients, families, and healthcare systems [4, 5, 6]. Myocardial fibrosis represents an inevitable pathological pathway through which persistent inflammation and cardiac tissue injury propagate and culminate in heart failure [7, 8]. It is characterized by the excessive accumulation of extracellular matrix (ECM) components within the myocardial interstitium [9, 10, 11, 12]. This process is driven by the activation of quiescent cardiac fibroblasts (CFs), their phenotypic transdifferentiation into myofibroblasts (CMFs), and the subsequent excessive secretion of ECM components [13, 14, 15, 16]. Local myocardial inflammation and injury serve as initiating factors for fibrosis [17]; however, targeting inflammation alone is insufficient to mitigate fibrotic progression [18]. Notably, in certain contexts, inflammatory factors are even indispensable for the reversal of fibrotic lesions [4]. Investigating the mechanisms underlying myocardial fibrosis regression—particularly the phenotypic transitions between CFs and CMFs as well as their regulation by the inflammatory-immune microenvironment—holds significant potential for combating cardiac remodeling and heart failure [19, 20, 21].

Recent studies have demonstrated that the inflammatory microenvironment induces cellular phenotypic transitions through epigenetic regulatory mechanisms [22], which may drive the transformation of CFs to CMFs [23]. Specifically, proinflammatory cytokines such as tumor necrosis factor-α (TNF-α) and interleukin-6 (IL-6) can trigger cellular phenotypic transitions via epigenetic modifications—including DNA methylation, histone modifications, and non-coding RNA-mediated regulation—and thereby contribute to pathological processes such as tissue fibrosis and tumor metastasis [24, 25, 26, 27]. For instance, pro-inflammatory signals activate DNA methyltransferases (DNMTs) or histone deacetylases (HDACs) to silence tumor suppressor genes or epithelial markers (e.g., E-cadherin), while promoting mesenchymal phenotypes (e.g., N-cadherin, Vimentin), driving epithelial-mesenchymal transition (EMT) [28, 29, 30, 31]. Inflammation-related microRNAs (miRNAs) (e.g., miR-21, miR-155) amplify profibrotic signals by targeting key epigenetic molecules [32].

Epigenetics, which explores changes in gene expression without altering the DNA sequence, is a crucial link between the environment and the development of fibrosis [33, 34, 35, 36, 37, 38]. As an important RNA epigenetic modification, 5-methylcytosine (m5C) methylation occurs at the fifth carbon of cytosine in RNA molecules [39], widely present in mRNA, tRNA, rRNA, and non-coding RNA [40, 41, 42, 43, 44, 45]. It participates in both physiological and pathological processes by modulating RNA stability, subcellular localization, and translation efficiency [46, 47], as well as regulating nuclear mRNA export and protein translational processes [48]. Within the cardiovascular system, m5C methylation has been implicated in conditions such as myocardial hypertrophy and atherosclerosis [49]; however, its specific role in immune inflammation-mediated myocardial fibrosis remains elusive.

## 2. Literature Review

### 2.1 Characteristics and Regulatory Enzyme System of m5C Methylation Modification

m5C modification is primarily mediated by three categories of proteins: methyltransferases, demethylases, and recognition proteins (referred to as “writers”, “erasers”, and “readers”, respectively) [50, 51, 52]. As “writers”, m5C methyltransferases catalyze the formation of m5C through a methyl transfer reaction, utilizing S-adenosylmethionine (SAM) as the methyl donor to transfer a methyl group to the cytosine residue [53]. Demethylases, termed “erasers” (e.g., the ten-eleven translocation (TET) enzyme family), are responsible for mediating RNA demethylation [54]. In contrast, RNA m5C recognition proteins (i.e., “readers”) such as Alyref (RNA binding and export factor, REF) and Y-box binding protein 1 (YBX1) exert their biological functions by specifically recognizing and binding to m5C sites [54].

Currently known m5C methyltransferases mainly include members of the NOP2/Sun RNA methyltransferase family (NSUN1-7) and DNA methyltransferase 2 (DNMT2) [55, 56, 57]. These enzymes exhibit specific expression patterns in different tissues and cells, with distinct substrate preferences. For example, NSUN2 primarily modifies mRNA and non-coding RNAs, while NSUN6 shows a preference for tRNA modification [58]. Studies have confirmed that m5C RNA methyltransferases are closely associated with the occurrence and development of myocardial diseases [59]. Recent research has found that NSUN2-mediated m5C methylation modification can alleviate doxorubicin-induced cardiotoxicity [60]. The binding of NSUN2 to m5C is closely linked to cardiovascular diseases, and the NSUN2/p53 axis may serve as a potential mechanism for treating aging-related heart diseases [61].

m5C methylation modification exhibits dynamic and reversible characteristics, and its demethylation process may be catalyzed by TET family enzymes. The distribution of m5C modification is tissue-specific and developmental stage-specific, showing dynamic changes under different physiological and pathological conditions. High-throughput sequencing technologies have revealed that m5C modification sites are mainly concentrated in the coding regions and untranslated regions (UTRs) of RNA, potentially regulating gene expression by influencing RNA secondary structures, protein-RNA interactions, and other mechanisms [62, 63, 64, 65]. In mRNA, m5C sites are distributed throughout the genome and are most frequently located in C-G-rich regions. These sites primarily reside in the UTRs of mRNA, especially near the 3^′^UTR. The distribution of m5C sites in the coding sequence (CDS) remains undetermined. Some scholars suggest that m5C sites have the lowest density in CDS, while other studies indicate that m5C sites are also abundant in the downstream region of the translation initiation site.

The regulatory influence of m5C modification on RNA fate is largely determined by m5C readers, which critically regulate RNA export, stability, and translation initiation [66]. RNA m5C-binding proteins, such as Alyref and YBX1, are considered “readers” that exert biological effects by recognizing and binding to m5C sites. YBX1 is identified as a cytoplasmic mRNA m5C reader in human cells. Structural analysis of YBX1 reveals that it recognizes m5C in its cold shock domain through the indole ring of W65 [67, 68, 69, 70]. YBX1 specifically targets several m5C-containing oncogenes (e.g., Hepatoma-derived growth factor (HDGF)) and promotes their stability and subsequent cancer progression by recruiting ELAV-like RNA-binding protein 1 (ELAVL1), a well-known mRNA stability maintenance factor. YBX1 and its molecular chaperone Poly(A) Binding Protein Cytoplasmic 1 (PABPC1a) regulate maternal mRNA stability during the maternal-to-zygotic transition in zebrafish embryo development [71].

Another m5C reader is Alyref, a protein with a canonical RNA-binding function in the transcription-export complex (TREX). As the first identified nuclear “reader” protein that recognizes RNA m5C modifications, Alyref modulates the functions of m5C-modified RNAs [72, 73, 74, 75, 76]. Alyref primarily binds to the 5^′^ and 3^′^ regions of mRNA and is highly conserved from *Saccharomyces cerevisiae* to humans. It contains a conserved RNA-binding domain (RBD) and glycine/arginine-rich sequences at both the N-terminus (amino acids 24–94) and C-terminus (amino acids 205–238). Like other RBD structures, the exposed β-sheet surface of Alyref contains hydrophobic and charged residues, which mediate interactions with other biomacromolecules. Alyref specifically binds to m5C modification sites via lysine at position 171, thereby facilitating mRNA nuclear export.

### 2.2 Immunoinflammatory Response and Myocardial Fibrosis

The immunoinflammatory response constitutes a complex network-regulated process. In the early phase of cardiac injury, innate immune cells—including neutrophils and macrophages—rapidly infiltrate the injured site and initiate inflammation through the secretion of proinflammatory cytokines. Subsequently, adaptive immune cells (e.g., T lymphocytes and B lymphocytes) are activated to participate in inflammatory modulation. These immune cells contribute to the activation of CFs and the promotion of myocardial fibrosis by secreting a variety of proinflammatory cytokines (e.g., TNF-α, IL-1β, IL-6) and growth factors (e.g., TGF-β).

**Fig. 1.**
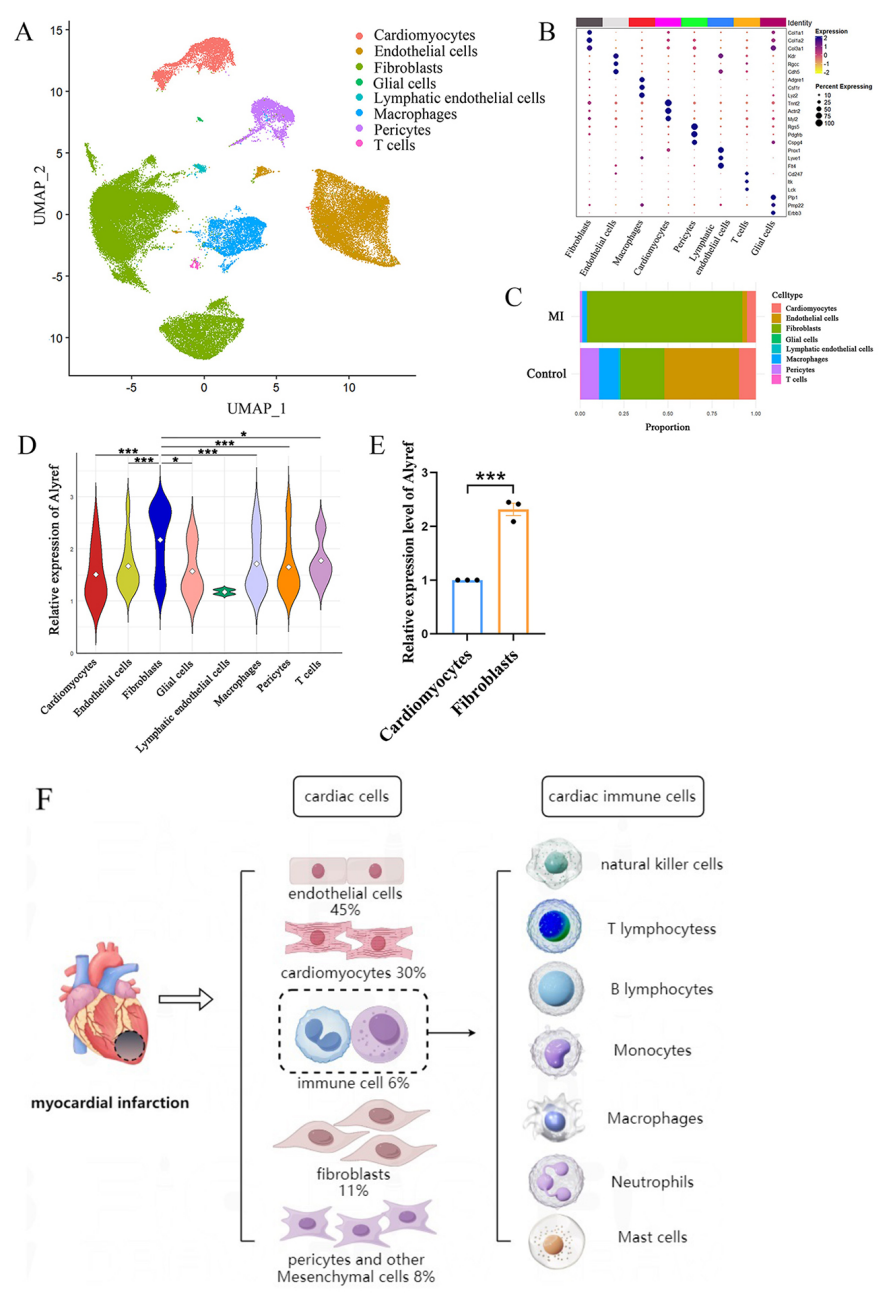
**Cell-type-specific landscapes of 5-methylcytosine (m5C) modifications in a mouse model of myocardial infarction**. (A) Single-cell sequencing analysis of the control group and myocardial infarction group. Uniform Manifold Approximation and Projection (UMAP) visualization of cardiac tissue cell types in the control and myocardial infarction (MI) groups. (B) Dot plots displaying mRNA levels and proportions for each specific cell population. (C) Bar chart illustrating the proportions of different cell clusters in the control and MI groups, with colors representing distinct major cell clusters. (D) The violin plot shows the expression of Alyref in each cell group. (E) Relative expression of m5C reader Alyref mRNA in hypoxia-induced cardiomyocytes and cardiac fibroblasts [82]. (F) Cellular composition of myocardial tissue and myocardial local immune cell types in myocardial infarction. * represents *p* < 0.05, ** represents *p* < 0.01, *** represents *p* < 0.001.

In myocardial injury, circulating macrophages and neutrophils are the first to secrete proinflammatory cytokines and growth factors, thereby regulating cardiac remodeling [77, 78, 79]. During this process, dendritic cells (DCs) mediate the recruitment of monocytes and macrophages. Recruited eosinophils and mast cells release mediators that contribute to coronary vasoconstriction, leukocyte recruitment, and scar formation. Within the adaptive immune response, effector T cells—particularly Th17 cells—drive the pathogenesis of cardiac fibrosis [80], whereas regulatory T cells (Treg cells), which exert protective effects, suppress and attenuate inflammatory responses [81] (Fig. 1, Ref. [82]).

The immuno-inflammatory system activates cardiac fibroblasts through a variety of unknown molecular mechanisms, driving immune-fibroblast crosstalk in human cardiac disease [83]. Locally activated CFs undergo proliferation and differentiate into CMFs, which are characterized by the upregulated expression of α-smooth muscle actin (α-SMA) and enhanced contractile capacity. These cells robustly synthesize and secrete ECM proteins, including collagen. Concurrently, the homeostatic balance between matrix metalloproteinases (MMPs) and their tissue inhibitors (TIMPs) is perturbed, resulting in excessive ECM deposition and the development of cardiac fibrosis. Sustained inflammatory responses drive the progressive exacerbation of fibrosis, ultimately culminating in severe pathological consequences such as cardiac diastolic dysfunction and arrhythmias.

### 2.3 Regulatory Role of m5C Methylation in Immunoinflammatory Response

Recent studies have demonstrated that m5C methylation modifications play critical roles in the development, differentiation, and functional regulation of immune cells [84]. In macrophages, m5C methylation modulates their polarization status by regulating the mRNA stability of key inflammation-associated genes [85]. For instance, NSUN2-mediated m5C modification stabilizes the mRNA of M1 macrophage-specific genes, thereby promoting the production of proinflammatory cytokines. Conversely, the absence of specific m5C modifications may induce polarization toward the anti-inflammatory M2 macrophage phenotype [86]. In T cells, m5C methylation is involved in regulating the expression of genes associated with the T cell receptor signaling pathway, which in turn influences T cell activation and differentiation. Studies have demonstrated that NSUN5 deficiency results in aberrant differentiation of CD4⁺ T cells into Th1 and Th17 subsets, thereby altering the magnitude and spectrum of inflammatory responses [87]. Furthermore, m5C methylation indirectly modulates immune cell-mediated inflammatory responses through modifications of non-coding RNAs, such as miRNA precursors. These findings indicate that m5C methylation may act as a key epigenetic regulatory mechanism in immunoinflammatory responses.

RNA methylation modulates immune cell activation and differentiation, which may impact their roles in cardiac inflammation and remodeling [88]. Han *et al*. [89] demonstrated that upregulated m5C modification is associated with neutrophil migration and granulocyte activation. Specifically, m5C modification directly influences macrophage polarization and induces the expression of proinflammatory cytokines, including granulocyte-macrophage colony-stimulating factor (GM-CSF), TNF-β, IL-1β, and IL-6, thereby promoting pulmonary fibrosis. Additionally, m5C-mediated upregulation of MMP9 and Lipocalin-2 (Lcn2) expression may also be critical for Particulate Matter 2.5 (PM2.5)-induced fibrosis in the lung during exposure to atmospheric pollution [89]. However, due to the complexity of RNA modifications and the diversity of immune cell subsets, the interaction network between RNA modifications and immune cells remains largely elusive, warranting further investigation [90].

### 2.4 Possible Mechanisms of m5C Methylation Modification Regulating Myocardial Fibrosis

#### 2.4.1 m5C Regulates Immune Cell Activation and Inflammatory Cytokine Production

During myocardial fibrosis, m5C methylation exerts regulatory effects through multiple mechanisms. Specifically, m5C methylation modifies RNAs of inflammation-associated genes in immune cells, thereby regulating the production and release of inflammatory cytokines and indirectly influencing cardiac fibroblast activation. Furthermore, m5C methylation directly modifies RNAs of fibrosis-related genes in cardiac fibroblasts—including collagen genes, α-SMA genes, and genes encoding components of the TGF-β signaling pathway—thereby affecting the stability and translational efficiency of these RNAs.

Studies have found that in fibrotic myocardial tissues, the expression of multiple m5C methyltransferases is aberrant, and the transcriptome-wide m5C modification profiles exhibit significant alterations [91]. The m5C modification levels of specific genes show positive or negative correlations with the degree of myocardial fibrosis. For example, the absence of m5C modification in the mRNA of certain antifibrotic genes mediated by NSUN2 may lead to enhanced degradation of these mRNAs, promoting the fibrotic process. Additionally, m5C methylation may form complex regulatory networks by modifying non-coding RNAs involved in fibrosis regulation, such as long non-coding RNA (lncRNA) and circular RNA (circRNA) [92]. While evidence in fibrotic diseases remains sparse, studies on lung tumors have shown that NSUN2 elevates the m5C modification level of circRREB1. Moreover, Alyref can recognize this m5C modification and regulate the nuclear export of circRREB1 in an m5C-dependent manner, thereby upregulating its expression.

#### 2.4.2 Transdifferentiation of Cardiac Myofibroblasts

Beyond m5C, accumulating evidence indicates that N6-methyladenosine (m6A) modification acts as a dynamic regulator of cardiac pathophysiology, particularly in fibroblast-to-myofibroblast differentiation. In previous studies, methyltransferase-like 3 (METTL3)-mediated m6A modification was proven to promote the transdifferentiation of CFs to myofibroblasts during irradiation. Their results revealed that m6A RNA methylation induced aberrant lung-resident mesenchymal stem cells (LR-MSC) differentiation into myofibroblasts via the METTL3/miR-21/Phosphatase and tensin homolog (PTEN) signaling pathway [93]. Li *et al*. [94] elucidated the causal relationship between METTL3-mediated m6A modification, autophagy, and fibroblast-to-myofibroblast differentiation. While research on the role of m5C in cell differentiation remains limited, several studies have confirmed that the m5C writer NSUN2 influences neural cell differentiation—supporting the notion that RNA m5C modification is involved in regulating cell differentiation. Specifically, they demonstrated that loss of NSUN2 impairs normal brain development by reducing the number of differentiated upper-layer neurons in the cortical plate [95]. It is anticipated that an increasing number of studies will validate the role of m5C methylation modification in cardiac fibroblast differentiation in the future.

#### 2.4.3 Collagen Metabolism

Collagen, the most abundant ECM component in the left ventricle, has long served as a hallmark indicator for assessing the severity of cardiac fibrosis [96, 97]. Dysregulated collagen metabolism plays a pivotal role in inflammation-mediated myocardial fibrosis, characterized primarily by an imbalance between collagen synthesis and degradation, which culminates in excessive ECM deposition [98]. In inflammatory microenvironments, proinflammatory factors (e.g., TNF-α, IL-1β, and TGF-β) activate cardiac fibroblasts, upregulate transcription of type I and III collagens, and enhance the expression of profibrotic genes via epigenetic modifications (e.g., DNA methylation and histone acetylation) [99]. Concurrently, inflammatory signals suppress the activity of MMPs and promote the production of TIMPs, thereby reducing collagen degradation [100]. Additionally, reactive oxygen species (ROS) and inflammation-mediated oxidative stress further exacerbate collagen cross-linking, forming irreversible fibrotic networks that ultimately increase myocardial stiffness and deteriorate cardiac function [101].

**Fig. 2.**
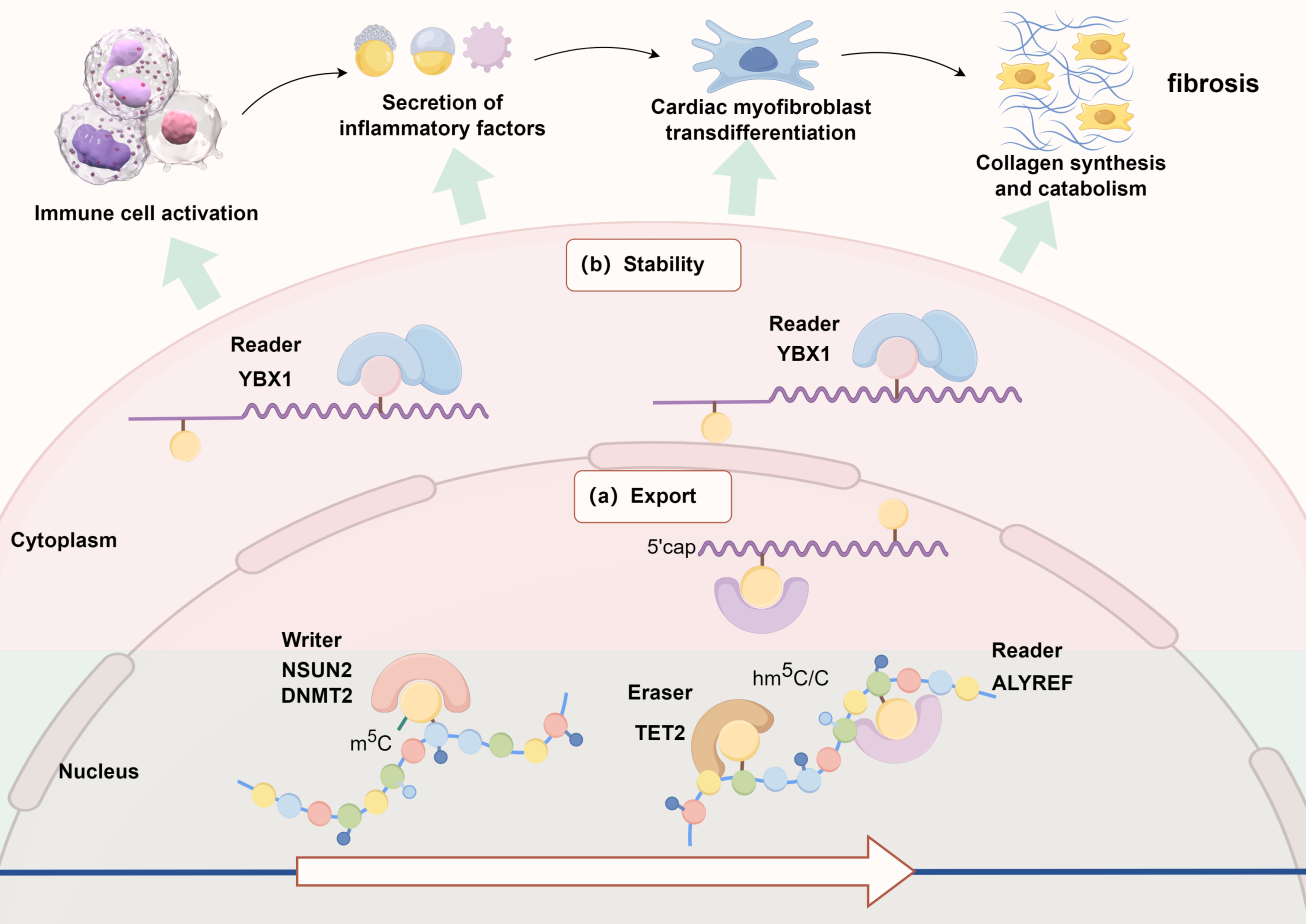
**Schematic diagram of the possible mechanism of m5C methylation modification in the regulation of myocardial fibrosis**. YBX1, Y-box binding protein 1; NSUN2, NOP2/Sun RNA methyltransferase family 2; DNMT2, DNA methyltransferase 2; TET2, ten-eleven translocation 2.

The role of RNA m5C methylation modification in myocardial collagen metabolism remains elusive (Fig. 2). Our preliminary studies have demonstrated that the m5C reader Alyref is upregulated in activated cardiac fibroblasts during the early phase after myocardial infarction (MI). The differential expression pattern of Alyref across cell populations suggests its potential dual role as a biomarker for fibroblast activation and a therapeutic target in cardiac repair mechanisms. Our findings indicate that Alyref knockdown significantly downregulates the expression of collagen and elastin, while also reducing collagen cross-linking density and ECM stiffness. In our study, Fibulin-1 (Fbln1) and Lysyl Oxidase-Like 1 (Loxl1) were identified as functional targets of Alyref; protein interaction analyses revealed their close association with collagen and elastin. Fbln1, a secreted glycoprotein, facilitates the stabilization and cross-linking of ECM proteins during collagen deposition. Loxl1 oxidizes lysine and hydroxylysine residues on collagen and elastin chains into highly reactive aldehydes, thereby forming inter- and intra-chain covalent cross-links to regulate cardiac remodeling. The differential expression pattern of Alyref across cell populations implies its potential dual role as a biomarker for fibroblast activation and a therapeutic target in cardiac repair mechanisms. Our results demonstrated that Alyref knockdown significantly downregulated the expression of collagen and elastin, while concurrently reducing collagen cross-linking density and ECM stiffness.

**Fig. 3.**
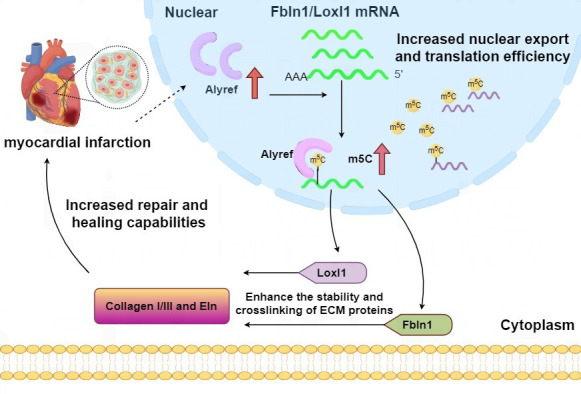
**Possible mechanisms by which local Alyref regulates collagen production in myocardium after myocardial infarction**. Explanation: the first red upward arrow means injuries such as myocardial infarction would stimulate the expression of Alyref. The second red upward arrow means Alyref promotes the m5C modification. Fbln1, Fibulin-1; Loxl1, Lysyl Oxidase-Like 1; Eln, Elastin; ECM, extracellular matrix.

In our study, Fbln1 and Loxl1 were identified as functional targets of Alyref; protein interaction analyses revealed their intimate association with collagen and elastin (Fig. 3). Fbln1, a secreted glycoprotein, facilitates the stabilization and cross-linking of ECM proteins during collagen deposition. Loxl1 oxidizes lysine and hydroxylysine residues on collagen and elastin chains into highly reactive aldehydes, thereby forming inter- and intra-chain covalent cross-links to modulate cardiac remodeling. Fbln1 overexpression was found to partially reverse the inhibitory effect of Alyref knockdown on Loxl1 expression, as well as on collagen and elastin synthesis. Thus, the Fbln1/Loxl1 axis may function as a downstream pathway of Alyref in regulating collagen metabolism and ECM protein synthesis following MI [82]. Single-cell sequencing data reveal that fibroblasts are the dominant cell population in myocardial tissue after MI. We further demonstrated that m5C readers, particularly Alyref, are most prominently expressed in activated cardiac fibroblasts. RNA immunoprecipitation sequencing (RIP-seq) analyses confirmed that Alyref modulates the synthesis of ECM proteins, including collagen and elastin, in cardiac fibroblasts [82].

### 2.5 Potential Therapeutic Roles of Targeting m5C Methylation Modification in Myocardial Fibrosis

Based on the critical role of m5C methylation in immune-inflammatory-mediated myocardial fibrosis, targeted regulation of m5C methylation may emerge as a novel strategy for treating myocardial fibrosis. However, this field still faces numerous challenges, such as the specificity of m5C methylation regulation. Drug delivery systems should be optimized and overcome the challenges in achieving cardiac tissue specificity for drugs (e.g., small-molecule modulators of NSUN2 or Alyref). The use of nanoparticles for drug delivery in most cases substantially enhances drug efficacy, improves pharmacokinetics and drug release, and limits their side effects. However, further enhancement in drug efficacy and significant limitation of adverse side effects can be achieved by specific targeting of nanocarrier-based delivery systems, especially in combination with local administration, which would enhance translational relevance [102]. Future research is required to more precisely dissect the spatiotemporal-specific regulatory network of m5C methylation in myocardial fibrosis and develop more specific and safe intervention strategies. Combined with existing antifibrotic therapeutic strategies, m5C methylation-targeted therapy may offer new hope for patients with myocardial fibrosis.

### 2.6 Prospects

As a pivotal epigenetic regulatory mechanism, m5C methylation modification orchestrates the immune microenvironment of myocardial fibrosis by governing immune cell activation, inflammatory cytokine secretion, and extracellular matrix remodeling. Elucidating the molecular mechanisms of m5C methylation in this process not only deepens our understanding of myocardial fibrosis pathogenesis but also furnishes a theoretical foundation for developing novel diagnostic biomarkers and therapeutic targets. Future research should be strategically focused on:

(1) Mapping the spatiotemporally specific m5C methylation modification landscape during myocardial fibrosis. Understanding the mechanisms of inflammation-mediated myocardial fibrosis requires a systematic assessment of cell types and their spatial organization, connectivity, and functional properties. The combined method of single-cell m5C sequencing and spatial transcriptomics is expected to create a spatially resolved and functionally aware cell map of the myocardial fibrosis region. These methods help identify major cell classes and cell subsets with relevant gene expression profiles. Single-cell transcriptomics and spatial transcriptomics technology are of great significance in life science research; the former can analyze cell heterogeneity, mine rare cells, and construct developmental trajectories from the single-cell level. The latter correlates transcription information with the spatial location of cells to reveal the spatial basis of tissue function.

These technologies have significantly expanded the scope of life science research, enabling in-depth exploration of cellular mysteries and the functional mechanisms of tissues. While they face numerous challenges in development, technological advancements, and multidisciplinary integration—such as synergies with artificial intelligence and machine learning—are poised to overcome these obstacles, drive deeper biological discoveries, facilitate extensive clinical translation, and yield more breakthroughs for life sciences and human health. Notably, their integration holds great promise for high-resolution exploration of the immune microenvironment. It promises to elucidate dynamic changes in m5C modification profiles at different stages of fibrosis using single-cell sequencing and spatial transcriptomics. Identify key m5C-modified RNAs in immune cells, fibroblasts, and cardiomyocytes that drive fibrotic progression.

(2) Revealing the crosstalk between m5C methylation and other epigenetic modifications. Tu *et al*. [103] found that demethylases, ALKBH3, exert a pro-fibrotic effect in pathological skin fibrosis by reshaping m6A RNA modification patterns. Their observation bridges the understanding of the link between m1A and m6A methylation, the two fundamental RNA modifications, underscoring the participation of “RNA methylation crosstalk” in pathological events. However, research specifically focusing on myocardial fibrosis remains lacking. Crosstalk between m5C methylation modifications and other RNA modifications has also not been reported yet. This is the direction of future research [103].

(3) Design small-molecule inhibitors or activators of NSUN2, TET2, YBX1, and Alyref with high tissue specificity (e.g., targeting cardiac fibroblasts). Moving forward, several translational priorities merit focused investigation to advance m5C methylation-based strategies for myocardial fibrosis. The development of nanocarrier-mediated delivery systems should be prioritized to enhance tissue-specific targeting of m5C-modulating therapies, thereby minimizing off-target effects on non-cardiac organs. Concurrently, systematic exploration of m5C methylation modifications as potential diagnostic and prognostic biomarkers for myocardial fibrosis is warranted. This includes comprehensive screening for m5C methylation signatures in peripheral blood or myocardial tissue that correlate with fibrosis severity. Critical to clinical translation will be rigorous validation of these candidate signatures in large, well-characterized patient cohorts, with the ultimate goal of enabling early detection of fibrotic progression and accurate prognosis prediction to guide personalized therapeutic interventions.

## 3. Conclusions

With the advancement of research, the m5C methylation regulatory network is anticipated to emerge as a novel breakthrough in the prevention and treatment of myocardial fibrosis. Integrating epigenetic mechanisms with precision medicine may offer innovative strategies for reversing cardiac remodeling and improving the prognosis of patients with heart failure. Nevertheless, this review has certain limitations. Most of the evidence cited herein is derived from *in vitro* models or animal studies (e.g., murine myocardial infarction models). Critical translational gaps remain, such as the lack of m5C signature data in human myocardial fibrosis samples.

## Data Availability

The raw data of m5C are deposited with Yan Hao and will be publicly available as of the date of publication. All data reported in this paper will also be shared upon request.

## Abbreviations

α-SMA, α-smooth muscle actin; CFs, cardiac fibroblasts; CMFs, cardiac myofibroblasts; DNMTs, DNA methyltransferases; ECM, extracellular matrix; EMT, epithelial-mesenchymal transition; HDACs, histone deacetylases; IL-6, Interleukin-6; m5C, 5-methylcytosine; MMPs, matrix metalloproteinases; NSUN, NOP2/Sun RNA; ROS, reactive oxygen species; TIMPs, Tissue Inhibitors of Metalloproteinases; TNF-α, Tumor Necrosis Factor-alpha; TREX, transcription export complex.
